# Machine learning-based algorithm identifies key mitochondria-related genes in non-alcoholic steatohepatitis

**DOI:** 10.1186/s12944-024-02122-z

**Published:** 2024-05-08

**Authors:** Longfei Dai, Renao Jiang, Zhicheng Zhan, Liangliang Zhang, Yuyang Qian, Xinjian Xu, Wenqi Yang, Zhen Zhang

**Affiliations:** Department of General Surgery, The First Affiliated Hospital of Anhui Medical University, 218 Jixi Road, Hefei, 230022 Anhui Province China

**Keywords:** Machine learning, Mitochondria, NASH

## Abstract

**Background:**

Evidence suggests that hepatocyte mitochondrial dysfunction leads to abnormal lipid metabolism, redox imbalance, and programmed cell death, driving the onset and progression of non-alcoholic steatohepatitis (NASH). Identifying hub mitochondrial genes linked to NASH may unveil potential therapeutic targets.

**Methods:**

Mitochondrial hub genes implicated in NASH were identified via analysis using 134 algorithms.

**Results:**

The Random Forest algorithm (RF), the most effective among the 134 algorithms, identified three genes: Aldo–keto reductase family 1 member B10 (*AKR1B10*), thymidylate synthase (*TYMS*), and triggering receptor expressed in myeloid cell 2 (*TREM2*). They were upregulated and positively associated with genes promoting inflammation, genes involved in lipid synthesis, fibrosis, and nonalcoholic steatohepatitis activity scores in patients with NASH. Moreover, using these three genes, patients with NASH were accurately categorized into cluster 1, exhibiting heightened disease severity, and cluster 2, distinguished by milder disease activity.

**Conclusion:**

These three genes are pivotal mitochondrial genes implicated in NASH progression.

**Supplementary Information:**

The online version contains supplementary material available at 10.1186/s12944-024-02122-z.

## Introduction

Non-alcoholic steatohepatitis (NASH) is characterized by the deposition of hepatic lipids, inflammatory responses, hepatocellular necrosis, and fibrosis [1]. NASH is a major contributor to end-stage liver disease globally [2] due to its complex pathophysiology [3–6]. Given its association with severe liver conditions and metabolic disorders [7–9], research on NASH is imperative. While liver biopsy serves as the benchmark for diagnosing NASH [10], its invasiveness and associated risks have led to poor patient acceptance [11], particularly considering the global increase in NASH prevalence [10, 12]. Due to its invasiveness, susceptibility to sampling and observer variations, and impracticality for a population of up to one billion individuals worldwide, liver biopsy is inadequate [13]. An urgent need exists for non-invasive diagnostic markers for NASH. Treatment remains challenging owing to the absence of approved specific drugs [14], highlighting further the importance of identifying potential therapeutic targets.

A previous study reported a correlation between endoplasmic reticulum stress and mitochondrial dysfunction in pathogenesis [15]. The transmembrane 6 superfamily member 2 located in the endoplasmic reticulum regulates lipid metabolism and is associated with the advancement of non-alcoholic fatty liver disease (NAFLD) [16, 17]. However, the precise contribution of mitochondrial dysfunction to NAFLD pathogenesis remains unclear. Mitochondria are essential for cellular function, generating energy through oxidative and phosphorylation processes [18]. In patients with NASH, mitochondrial function is often compromised because of excessive fat oxidation and oxidative imbalance [19], resulting in mitochondrial impairment, thus worsening the pathophysiology of NASH [20, 21]. Numerous studies have documented abnormalities in mitochondrial structure and function in patients with NASH, including reduced mitochondrial respiratory chain activity, decreased adenosine triphosphate levels, elevated free fatty acid synthesis, and increased oxidative stress [22–26]. As NASH progresses, mitochondrial adaptability diminishes, resulting in suppressed function and the accumulation of damaged mitochondria [27]. Additionally, increased cholesterol synthesis and lipid peroxidation further damage mitochondrial function [28].

Recognizing the essential mitochondrial genes associated with NASH progression is crucial, as it may unveil potential therapeutic targets. The novel aspects of the study are the formulation of a NASH prediction model with the selected pivotal genes and the classification of NASH patients for non-invasive diagnosis and targeted therapy of NASH.

## Methods

### Analyzing Gene Expression Omnibus (GEO) data

Eight liver and one blood sample datasets related to NASH were obtained from the GEO database. Every dataset underwent processing with its corresponding platform files (Supplementary Table 1). Samples from GSE135251 and GSE48452 were merged to create a training cohort (merged cohort), and the batch correction method, ComBat, was applied to the combined dataset simultaneously. Subsequently, a principal component analysis (PCA) was conducted. The remaining seven cohorts were used as validation cohorts. Eight liver datasets were merged to create another validation set (meta-cohort), and the ComBat method was simultaneously applied to this merged dataset.

### Choosing mitochondria-related genes (MRGs)

In the Merge-Cohort, differentially expressed genes (DEGs) underwent filtration based on an absolute value of log(FC) > 0.5 and an adjusted *P*-value < 0.05. The MRGs were obtained from the MitoCarta database [29] and complemented with gene sets [30], as listed in Supplementary Table 2. DEGs were intersected with MRGs to identify mitochondria-related DEGs. Metascape [31, 32] and GeneMANIA [33] offer comprehensive bioinformatics analysis. These platforms aid in predicting gene function and analyzing potential biological pathways associated with the mitochondria, thereby revealing their biological significance.

### Identifying core MRGs and constructing a mitochondrial model

Twelve machine-learning algorithms were selected. Each algorithm was paired, resulting in 134 combinations, with one focused on variable selection and the other on predictive model development. In these pairs, the former screens the variables, whereas the latter constructs predictive models. Using the training dataset (Merge Cohort), these 134 algorithms were applied to identify crucial genes among the 15 MRGs and to develop predictive models using these genes.

### Biological mechanisms and immunological signatures within NASH

Gene set variation enrichment analysis (GSVA) can scrutinize gene expression and evaluate alterations in specific pathways, functionalities, or gene collections [34]. In the NASH group, GSVA identified enriched pathways. To examine alterations in the immunological signatures within NASH, 13 immune functions, and 22 immune cell signatures were obtained [35, 36]. This analysis determined the potential differences in the immune landscape between two groups.

### Exploring the immune landscape of genes

GSVA indicated the significant enrichment of various pathways that were influenced by the model genes. The exploration of the potential functions of these model genes encompassed the areas of inflammatory infiltration, lipid transportation, fatty acid metabolism, and immunological signatures.

### Single-cell profiling exploration

The dataset GSE129516 was acquired. First, the cohort was standardized with the “Seurat” package. Subsequently, the samples were divided into clusters based on the cell type. Following this, functional and cellular annotations were performed using the “Single R” package. Clusters were constructed for cellular reclassification based on immune cell markers.

### Categorizing individuals suffering from NASH

A consensus clustering analysis was used to categorize patients with NASH into distinct subgroups. Differences among various groups were compared to assess the extent of inflammatory infiltration, lipid accumulation status, severity of liver fibrosis, immune cells, and biological pathways across clusters. Weighted gene co-expression network analysis (WGCNA) [37] was conducted by establishing an appropriate soft threshold to screen DEGs between subgroups.

### Verification at the mRNA level and protein level

Information regarding the reagents used in this experiment and their suppliers is provided in Supplementary Table 3. Six liver specimens were obtained from patients with normal weight, and six were collected from patients diagnosed with obesity. Liver specimens from normal-weight patients and patients with obesity were fixed, embedded, and sectioned. Specimens from normal-weight patients showed no lesions, whereas all specimens from patients with obesity were diagnosed with NASH. RNA was extracted from the samples, followed by cDNA synthesis. The cDNA was then quantified. Expression levels of the target genes were determined and compared with t-tests. The primer sequences are located within Supplementary Table 4.

**Fig. 1:**
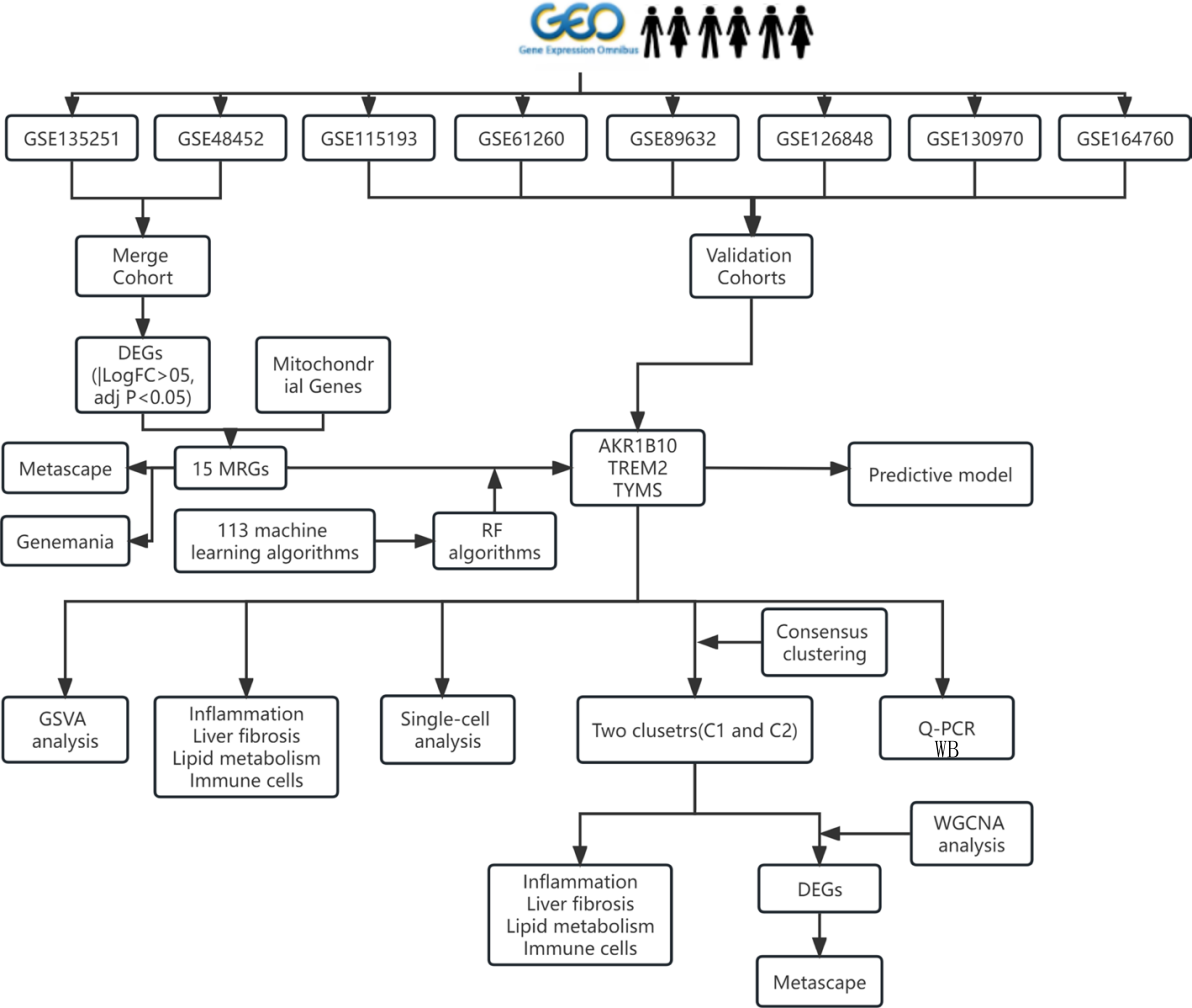
Flowchart of the design idea of this study

Four normal liver specimens and four NASH specimens were selected for protein extraction and western blotting (WB), respectively. Antibodies against *AKR1B10* and *TYMS* and the internal control β-actin antibody were sourced from Abcam (UK). Initially, 20 mg of liver tissue frozen in liquid nitrogen was obtained from each sample and mixed with pre-cooled steel beads and lysis buffer. Subsequently, the tissues were homogenized using a tissue homogenizer at 60 Hz for 120 s to ensure thorough grinding. Upon homogenization, the steel beads were eliminated, and the protein lysate was transferred to a separate centrifuge tube, then placed on ice for 30 min to ensure thorough tissue lysis. Following lysis, the supernatant was extracted via ultracentrifugation. Protein concentrations were assessed. A 10% separation gel was selected based on the size of the target protein molecules. Proteins underwent gel electrophoresis and were then transferred to a membrane utilizing the wet transfer method. Subsequently, the membrane underwent washing and blocking with tris-buffered saline containing tween and 5% skim milk. Following this, the specimen was subjected to overnight incubation with the primary antibodies at 4 °C, after which it underwent rinsing and subsequent incubation with the secondary antibodies. A schematic representation of the experimental procedure is depicted in Fig. 1.

## Results

### Statistics of samples

**Fig. 2:**
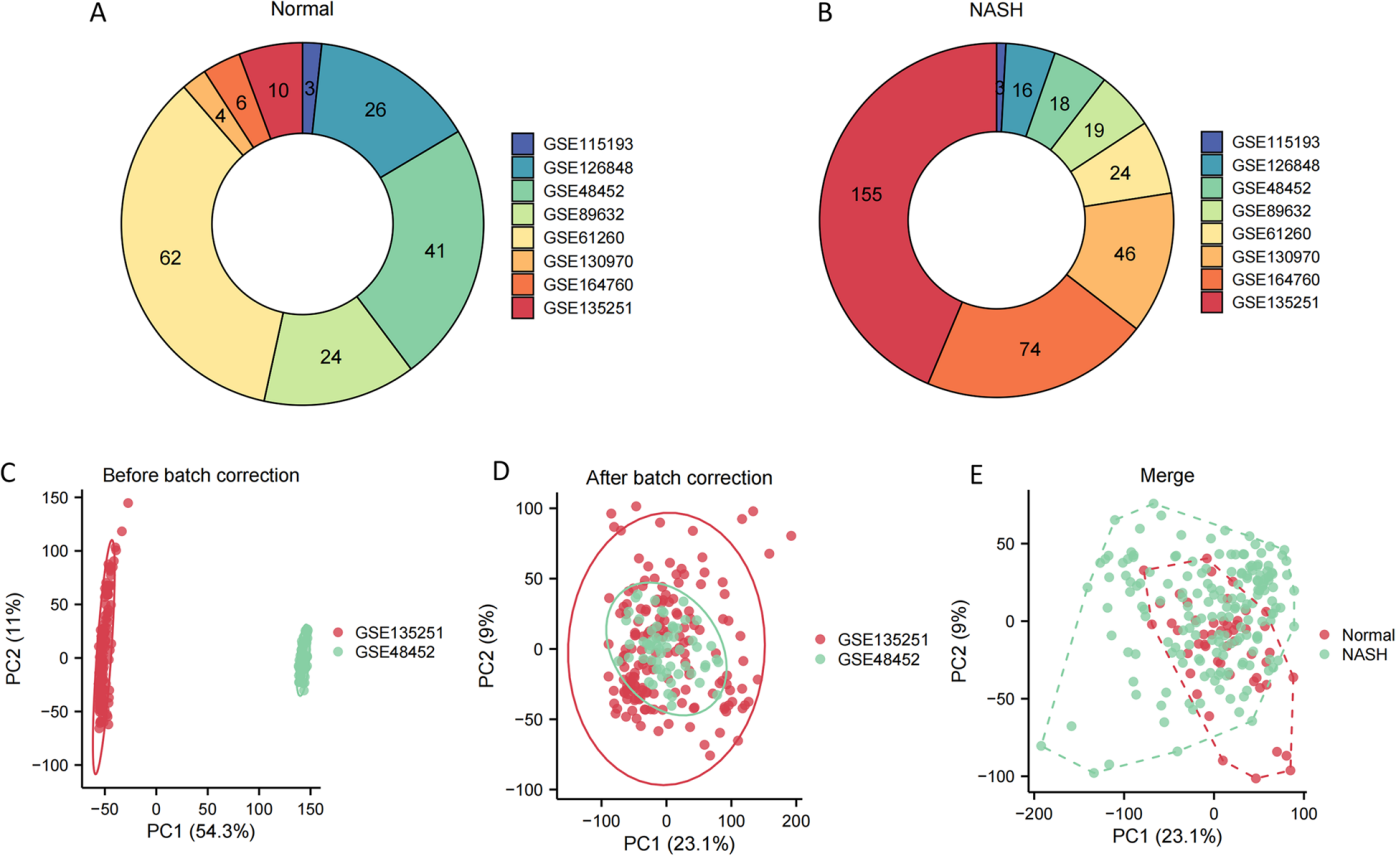
The handling for data. **A** The quantity of normal liver samples in each of the 8 GEO datasets. **B** The quantity of NASH specimens in each of the 8 GEO datasets. **C** Before the removal of batch effects, the PCA plot shows a distinction between samples from the two batches. **D** After eliminating batch effects, the PCA plot demonstrates the removal of batch effects, with samples from the two batches mixed together. **E** In the PCA plot, normal liver samples and NASH samples in the Merge-Cohort are distinguished

The number of normal liver specimens (Fig. 2A) and NASH specimens (Fig. 2B) included in the study from the 8 GEO datasets were represented in the donut chart. GSE135251 and GSE48452 were selected and merged into a new cohort termed Merge-Cohort, serving as the training set (Fig. 2C). Following the elimination of batch-related biases, samples from these two cohorts were effectively integrated (Fig. 2D). Moreover, within the new Merge-Cohort, normal liver samples and NASH samples were discernibly distinguished (Fig. 2E), affirming the inherent differences between these sample types.

### Fifteen MRGs related to the progression of NASH

**Fig. 3:**
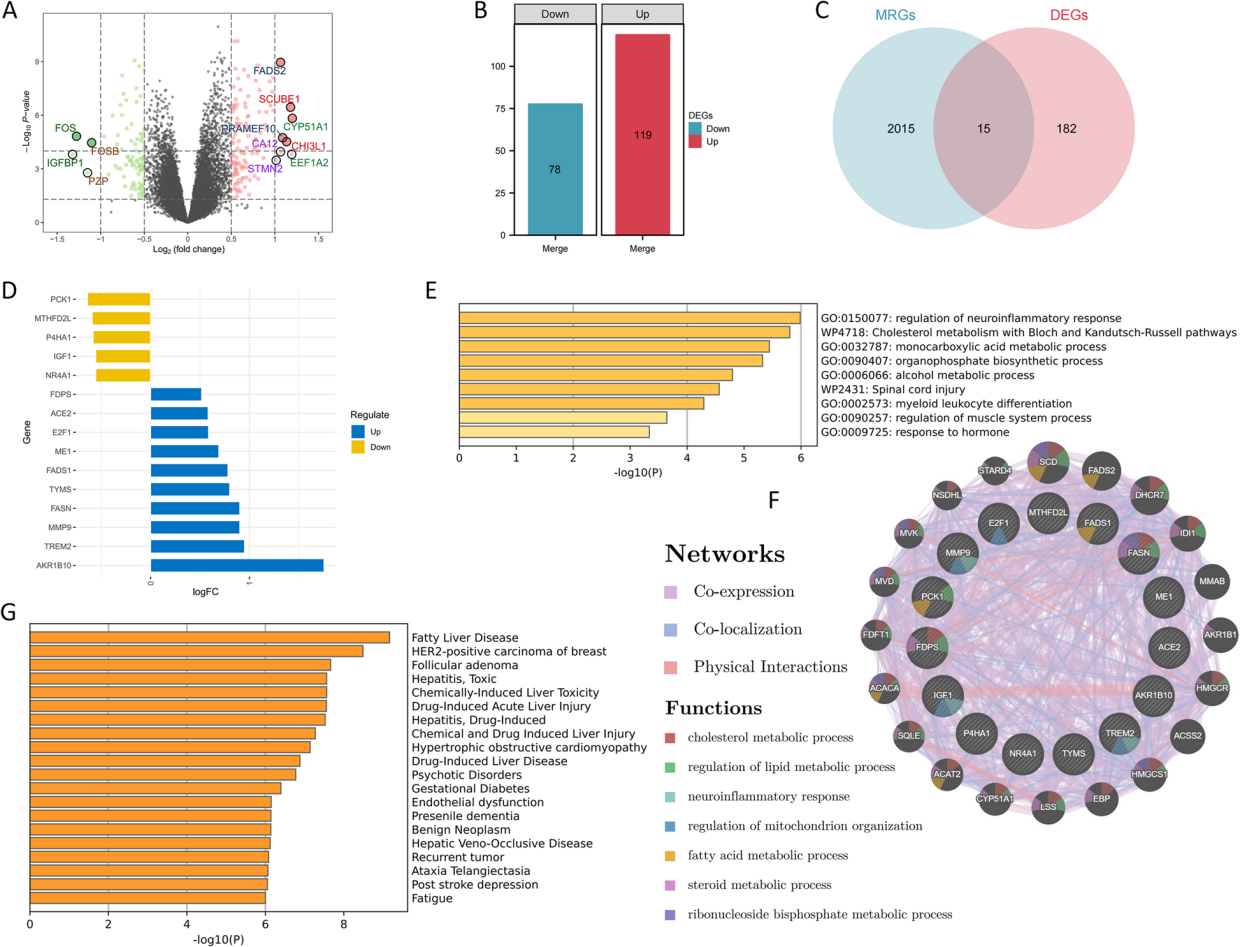
Discovery of 15 MRGs. **A**, **B** 197 DEGs were identified from the comparison between normal liver samples and NASH samples, comprising 78 downregulated genes and 119 upregulated genes. **C** Identification of 15 genes by the intersection of DEGs and MRGs. **D** Among these 15 genes, 5 genes were downregulated, and 10 genes were upregulated. **E** Nine biological pathways are related to the 15 genes. **F** The genes interacting with these 15 genes and the biological pathways they collectively involve. **G** The types of diseases affected by these 15 genes

Within the training dataset, 197 DEGs were distinguished between normal liver samples and those afflicted with NASH (Fig. 3A). In addition, 78 genes were under-expressed, whereas 119 showed the opposite trend (Fig. 3B). Through the intersection of 197 DEGs with 2,030 MRGs, 15 genes were identified (Fig. 3C). In the training dataset, five genes were downregulated, whereas ten genes displayed the opposite pattern (Fig. 3D). These 15 genes participate in diverse metabolic pathways, encompassing cholesterol metabolism, monocarboxylic acid metabolism, lipid metabolism, and mitochondrial tissue regulation (Fig. 3E and F). Additionally, these 15 genes are linked to numerous diseases, with the most pronounced correlation observed in NAFLD (Fig. 3G).

### Establishment of a predictive model encompassing three MRGs

**Fig. 4:**
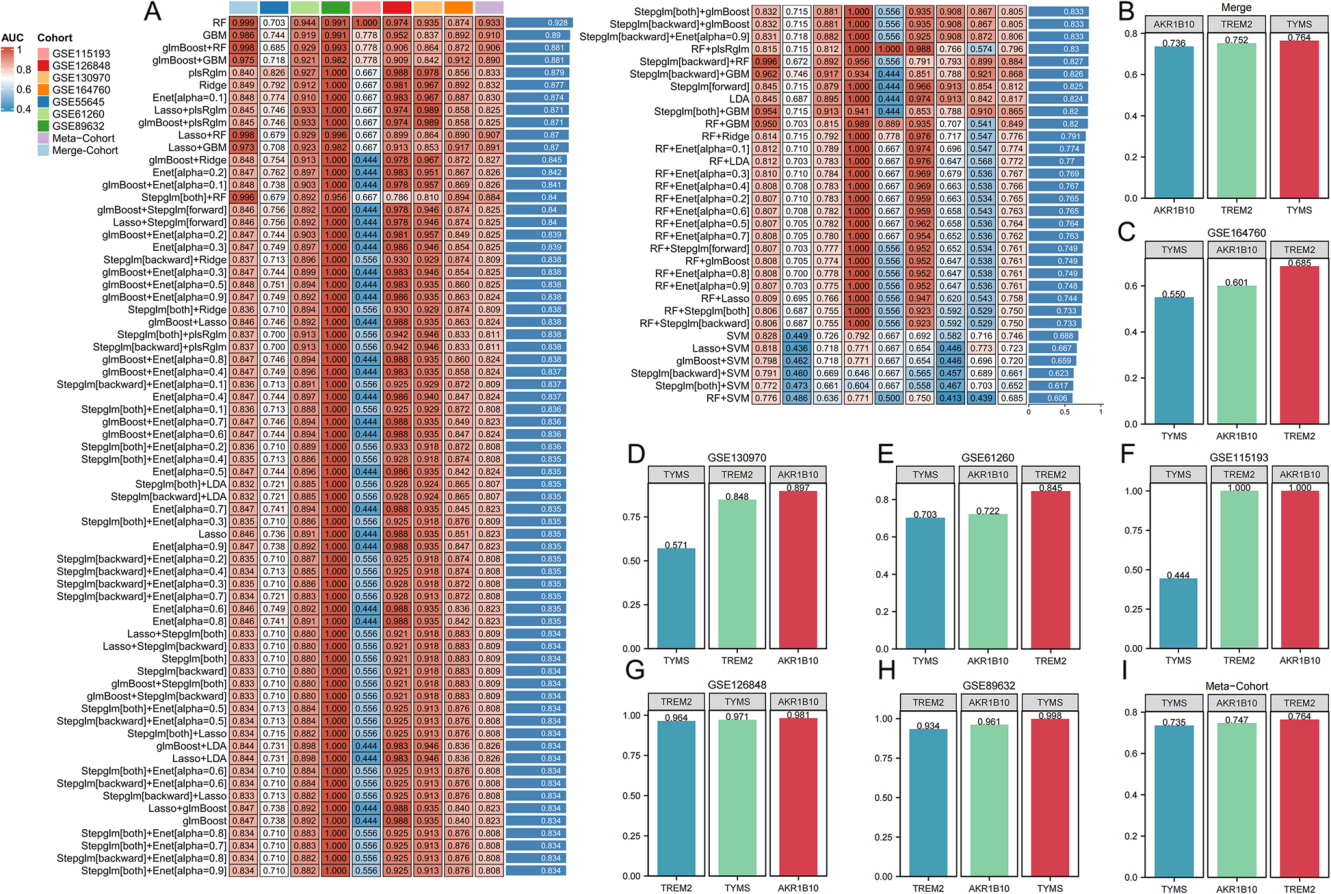
Machine learning techniques employed to formulate diagnostic models for NASH. **A** The model built with the RF algorithm demonstrated the highest predictive accuracy, boasting a C-index value of 0.928. **B**-**I** The AUC values of the three model genes for the separate diagnosis of NASH in both the training set and the external validation set were relatively high

Utilizing a combination of 12 algorithms, a total of 134 machine learning algorithms were generated. Following this, the 134 algorithms were employed to screen these 15 MRGs, aiming to establish a diagnostic model for NASH utilizing the selected genes. Among 134 algorithms, the RF algorithm exhibited the highest C-index value, and the predictive model constructed by the RF algorithm consisted of *AKR1B10*, *TYMS*, and *TREM2*. Using this model, the AUC values for diagnosing NASH patients in the training cohort and validation cohort (Merge-Cohort, GSE55645, GSE61260, GSE89632, GSE115193, GSE115198, GSE130970, GSE164760, meta-Cohort) were 0.999, 0.710, 0.942, 0.989, 1.000, 0.976, 0.913, 0.854, and 0.933, respectively (Fig. 4A). Additionally, the AUC values for diagnosing NASH patients using this model were higher than those of the model genes alone (Fig. 4B-I).

**Fig. 5:**
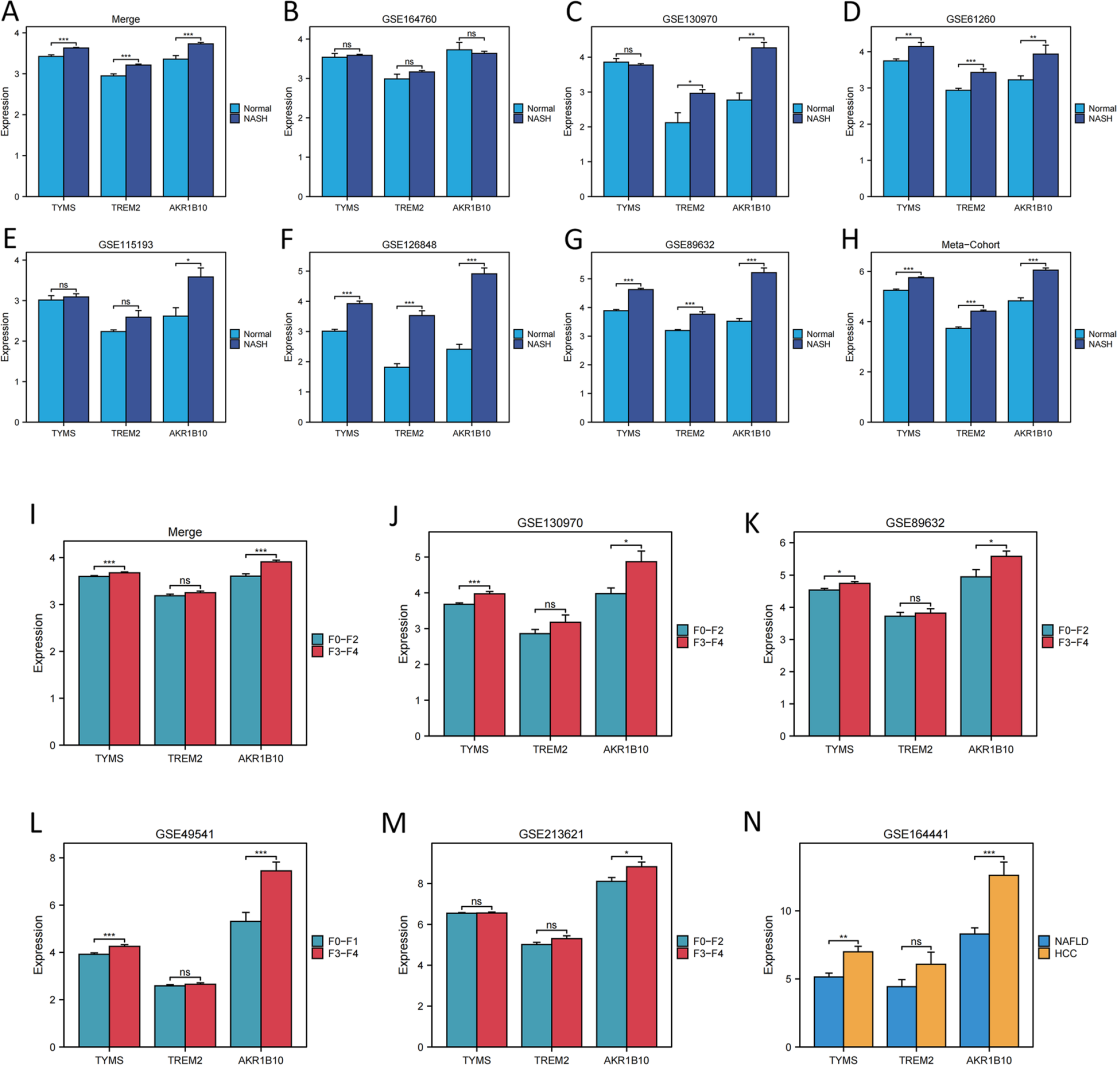
Analysis of three model genes based on their differential expression among various subgroups. (A-H) The three model genes were upregulated in NASH samples across the eight datasets. (I-M) *TYMS* and *AKR1B10* exhibited upregulation in the advanced-stage liver fibrosis phase across the five datasets*.* (N) In comparison to NAFLD, *TYMS* and *AKR1B10* exhibited significant upregulation in hepatocellular carcinoma samples linked with NAFLD. * *P* < 0.05, ** *P* < 0.01, *** *P* < 0.001

Furthermore, the three model genes were compared among different groups (normal vs. NASH group, F0-F2 vs. F3-F4 fibrosis group, and NAFLD vs. HCC group). Individuals with NASH displayed significantly higher levels of the three model genes than those in healthy individuals (Figs. 5A-H). Additionally, AKR1B10 and TYMS were associated with fibrosis advancement, exhibiting elevated expression levels in stages F3-F4 (Fig. 5I-IM). Furthermore, *AKR1B10* and *TYMS* were upregulated in hepatocellular carcinoma patients compared to NAFLD (Fig. 5N).

### The heightened expression of three MRGs implies a advanced stage of NASH

**Fig. 6:**
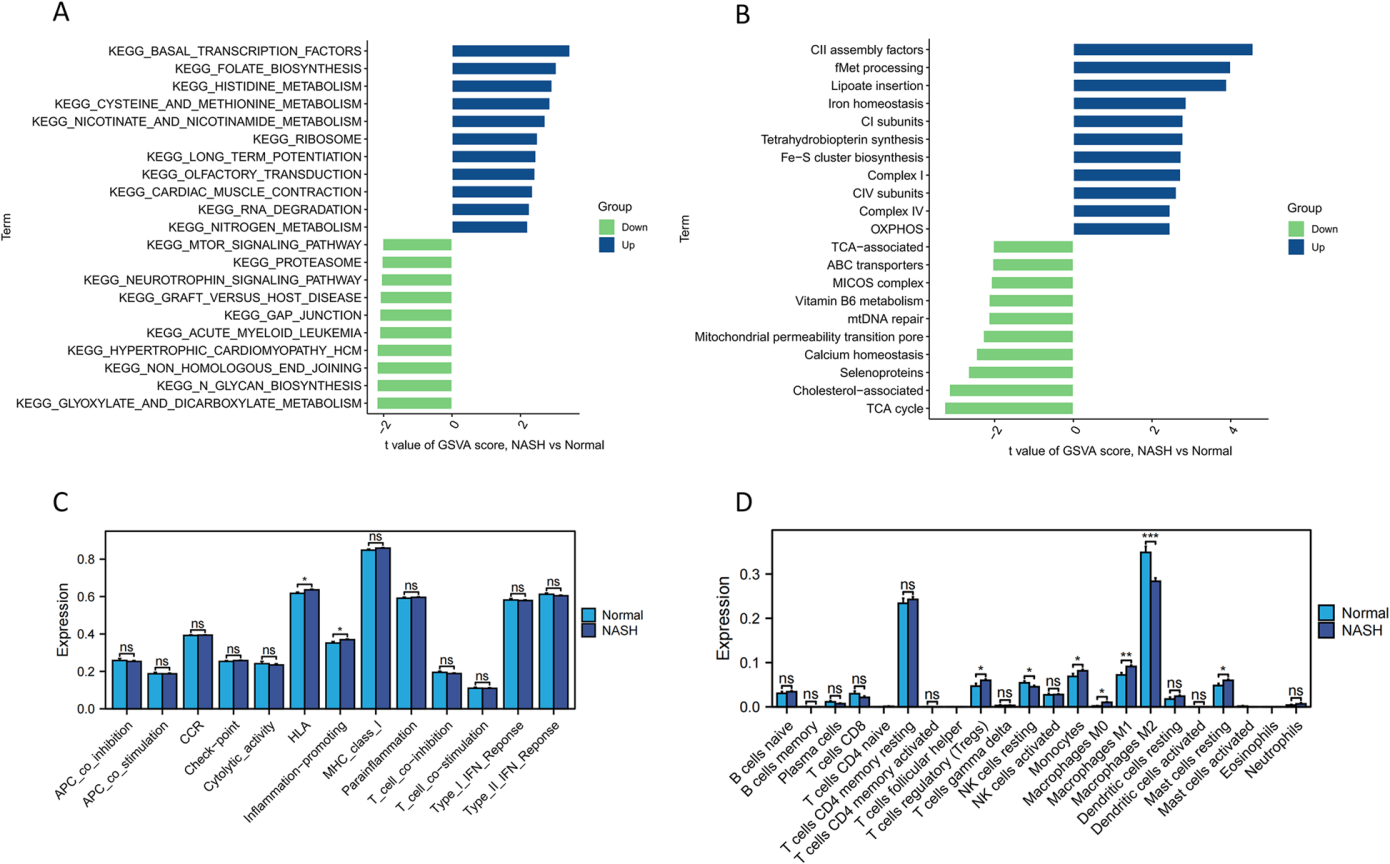
Distinct pathways and immune signatures between two groups. **A** Biological pathways altered in NASH are analyzed. **B** Mitochondrial-related pathways altered in NASH. **C** Two immune function scores were upregulated in the NASH group. **D** The abundance of M1 macrophages significantly increased in NASH, while M2 macrophages showed the opposite trend

Notable differences between patients with NASH and controls were observed in “nitrogen metabolism”, “cysteine and methionine metabolism”, and “nicotinate and nicotinamide metabolism”, all were upregulated in NASH (Fig. 6A). Additionally, mitochondrial pathways including “OXPHOS”, “complex IV”, and “Fe–S cluster biosynthesis” were upregulated (Fig. 6B). Across diverse biological processes, these signaling cascades are intricately linked to metabolic governance and oxidative stress. Furthermore, “HLA” and “inflammation-promoting” pathways were upregulated in NASH, indicating heightened inflammation promotion (Fig. 6C). The abundance of pro-inflammatory cytokine-producing “macrophages M1” was higher in the NASH group, whereas the number of anti-inflammatory cytokine-producing “macrophages M2” was diminished (Fig. 6D).

**Fig. 7:**
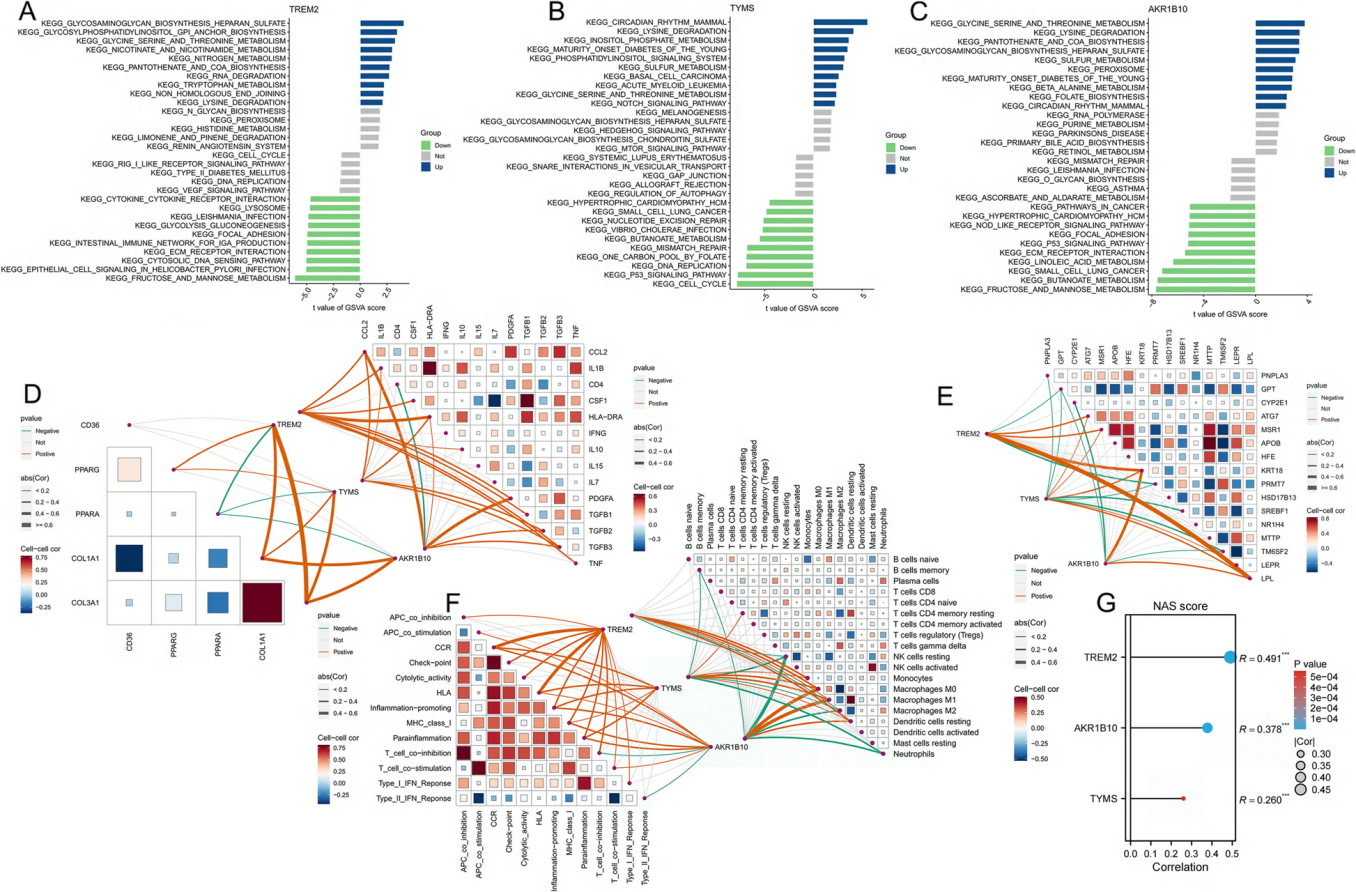
Association between *TREM2*, *TYMS*, and *AKR1B10* expression levels, and metabolism-related genes, and immune cell content in NASH. **A-C** The upregulation of the three genes was associated with the enrichment of specific biological pathways. **D** These three genes showed positive correlations with inflammation, lipid accumulation, and fibrosis, while exhibiting negative correlations with β-oxidation (*PPARA*). **E** Connections are evident between these three genes and the NASH-associated genes extracted from the GeneCard database, especially those genes with NASH relevance scores surpassing 10. **F** These three genes exhibited a positive correlation with pro-inflammatory immune function scores and the abundance of M1 macrophages, while demonstrating an inverse correlation with the abundance of M2 macrophages. **G** These three genes exhibited a significant positive correlation with NAS

The three MRGs were significantly heightened in pathways related to metabolic abnormalities such as “lysine degradation” and “glycine, serine, and threonine metabolism” (Figs. 7A-C). Furthermore, these genes exhibited significant and positive correlations with proinflammatory genes (*CCL2, IL1B, CSF1, HLA-DRA, IL10, PDGFA, TGFB1, TGFB2, TGFB3, and TNF*) as well as fibrotic genes (*COL1A1* and *COL3A1*) (Fig. 7D). Additionally, *TREM2* and *TYMS* demonstrated significant positive associations with the lipid synthesis gene (peroxisome proliferator-activated receptor gamma [*PPARG*]), whereas the three MRGs showed significant negative associations with genes related to peroxisome proliferator-activated receptor alpha [*PPARA*]. The three MRGs demonstrated a marked correlation with the highly ranked NASH genes contained in the GeneCards database (Fig. 7E). These three genes displayed a positive relationship with diverse immunological signatures, especially those related to inflammatory processes (Fig. 7F). The three MRGs showed significant associations with monocytes and macrophages, displaying positive correlations with M1 macrophages and negative correlations with M2 macrophages. Moreover, these three MRGs were positively linked with NAFLD activity score (NAS) (Fig. 7G).

### Mitochondrial attributes of the three MRGs

**Fig. 8:**
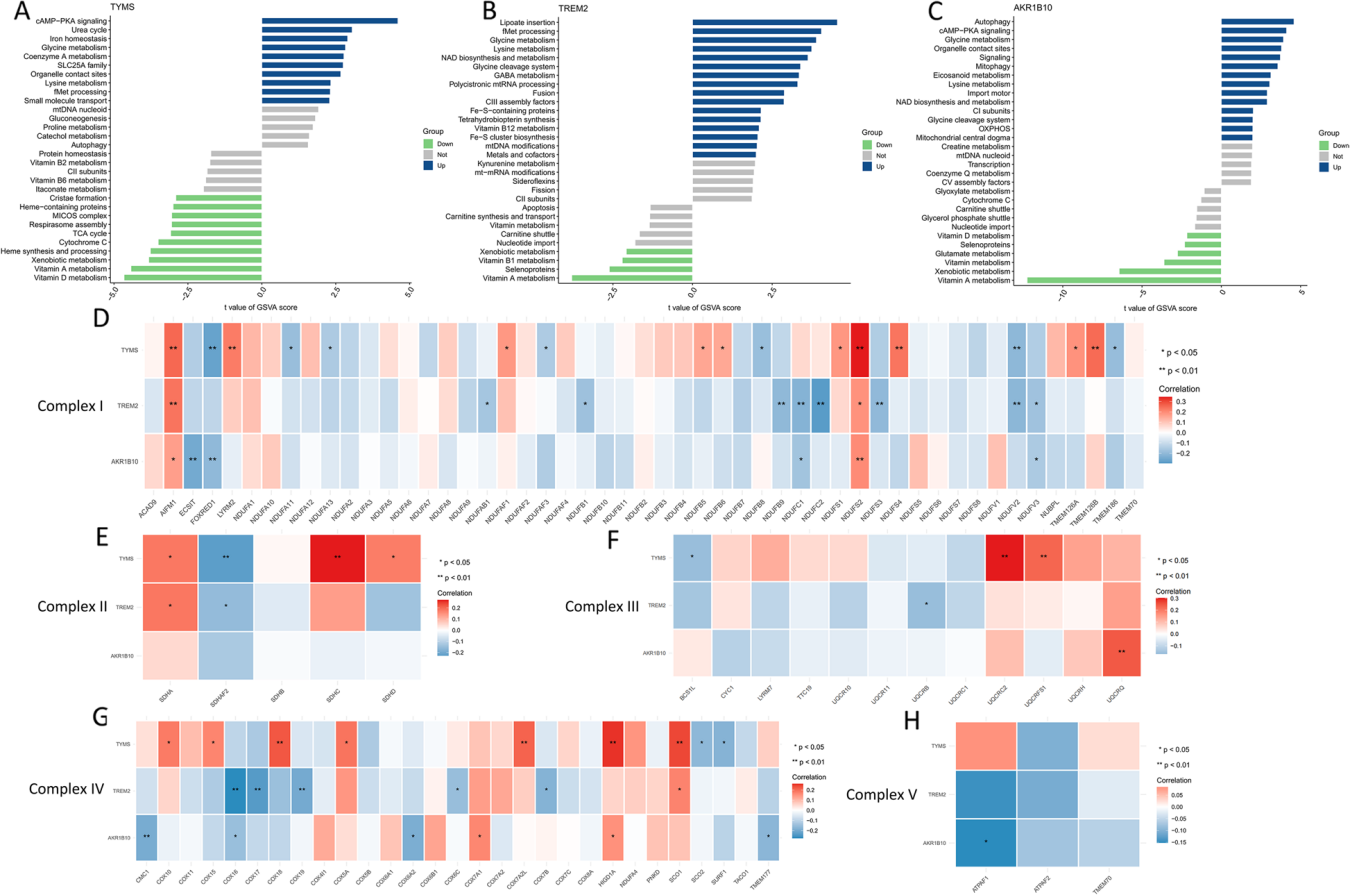
The attributes of mitochondria exhibited by three MRGs. **A** Based on *TYMS* expression levels, NASH was stratified into two groups, with the GSVA plot demonstrating significant enrichment of distinct mitochondrial-related pathways in each group. **B** Based on *TREM2* expression levels, NASH was stratified into two groups, with the GSVA plot demonstrating significant enrichment of distinct mitochondrial-related pathways in each group. **C** Based on *AKR1B10* expression levels, NASH was stratified into two groups, with the GSVA plot demonstrating significant enrichment of distinct mitochondrial-related pathways in each group. **D-H** Notable relationships between the three MRGs and the genes encoding the mitochondrial respiratory chain complexes, specifically complexes I through V

When these three MRGs are highly expressed in NASH, they exhibit significant enrichment in the “lysine metabolism” and “glycine metabolism” pathways (Figs. 8A-C). On the contrary, “glycine metabolism” pertains to the metabolic processes involving glycine and is linked to irregularities in hepatic lipid and carbohydrate metabolism. Moreover, the correlation of these three genes with genes associated with mitochondrial respiratory chain Complex I (I-V) in NASH (Figs. 8D-H) implies their potential contribution to NASH advancement by modulating mitochondrial function and metabolic irregularities.

### MRGs are abundant in M1 macrophage

**Fig. 9:**
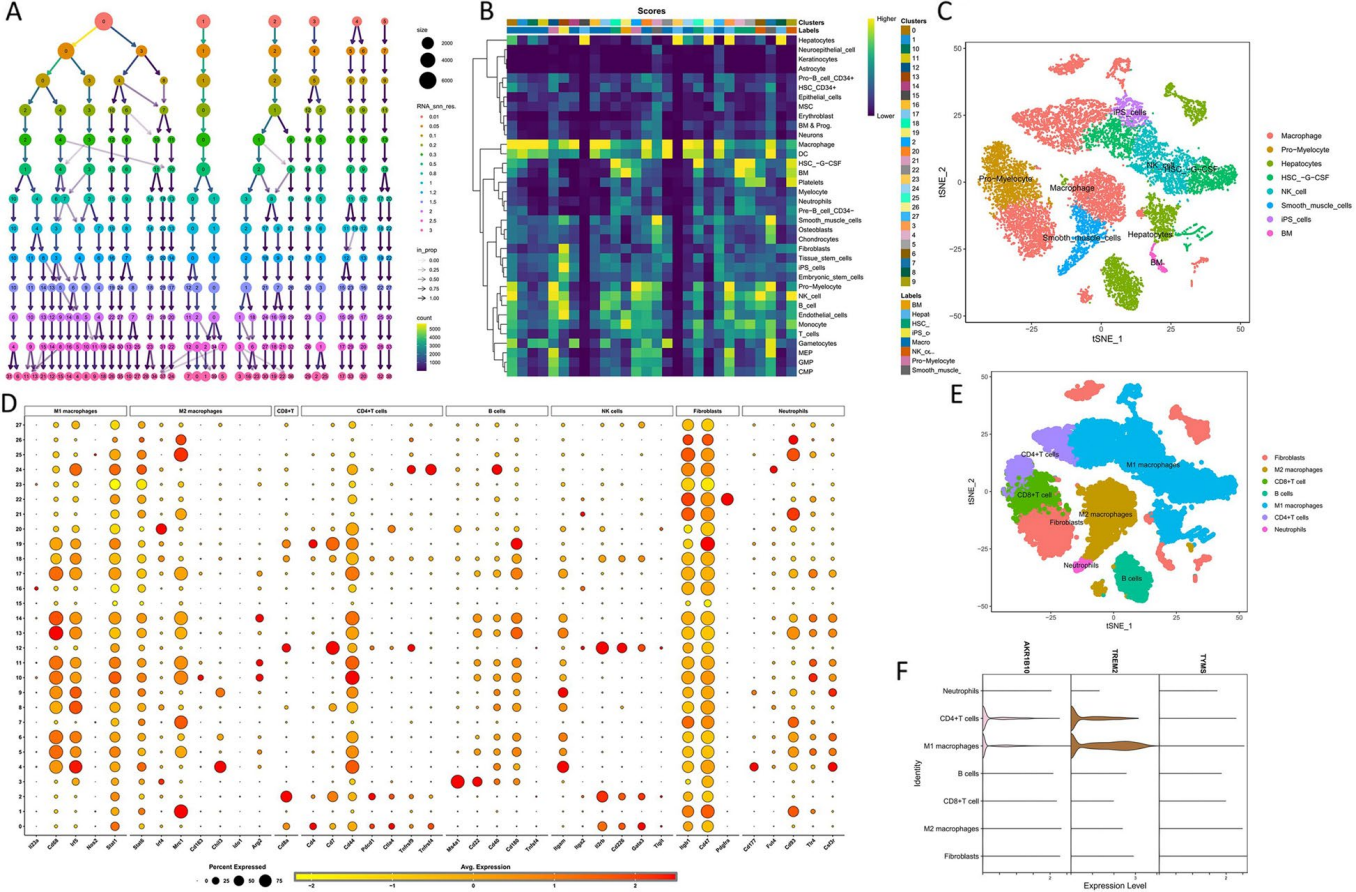
Single-cell analysis. **A** The dendrogram depicts the hierarchical clustering of the data into distinct clusters. **B** Automatic categorization of data into eight distinct cell types using the “Single R” package. **C** The distribution pattern of the eight identified cell types. **D** Expression profiles of seven immune cell surface markers across the 28 clusters. **E** The classification of data into seven immune cells. **F** *AKR1B10* and *TREM2* exhibit notable enrichment in M1 macrophages

From the GSE129516 dataset, 30,038 single cells were isolated. To streamline the analysis, dimensionality reduction was applied to the corrected data at a resolution of 1.5 (Fig. 9A). Following this, the single-cell data were segregated into 28 discrete clusters and automatically categorized into eight distinct cell types (Fig. 9B). The distribution patterns of the eight cell types are illustrated in Fig. 9C. Due to recognized limitations of the “Single R” package, a manual annotation process was initiated. Immune cell surface markers were utilized for the re-annotation of the single-cell data. Markers representative of the eight immune cell types are depicted in Fig. 9D. Following re-annotation, the single-cell data were categorized into M1 macrophages, M2 macrophages, fibroblasts, CD8 + T cells, CD4 + T cells, neutrophils, and B cells (Fig. 9E). *AKR1B10* and *TREM2* demonstrated significant overexpression in M1 macrophages, suggesting their involvement in inflammatory processes (Fig. 9F).

### Grouping individuals with NASH into two distinct categories

Through the expression profiles of the three model genes, individuals diagnosed with NASH were divided into two clusters, referred to as Cluster 1 and Cluster 2 (Supplementary Fig. 1A). Successful stratification of patients with NASH into distinct subgroups was validated using PCA (Supplementary Fig. 1B). The three MRGs were increased in the Cluster 1 subgroup (Supplementary Fig. 1C), whereas patients with NASH in the Cluster 2 subgroup demonstrated low expression levels of these genes. Furthermore, individuals belonging to Cluster 1 exhibited a higher occurrence of NAS and fibrosis stages ranging from F3 to F4 (Supplementary Fig. 1D and E). In the Cluster 1 subgroup, there was an increase in pro-inflammatory and fibrotic genes (Supplementary Fig. 1F). Moreover, there is an upregulation in the expression of the lipid synthesis gene (PPARG) in Cluster 1, whereas β-oxidation gene (*PPARA*) expression is downregulated. The “CCR”, “cytolytic activity”, “HLA”, “inflammation-promoting”, “MHC class I”, and “parainflammation”, exhibited significant upregulation in Cluster 1 (Supplementary Fig. 1G). The C1 subgroup displayed a higher abundance of neutrophils and M1 macrophages, whereas NK cells and M2 macrophages demonstrated an inverse trend (Supplementary Fig. 1H). Moreover, pathways related to inflammatory infiltration, such as “keg alanine aspartate and glutamate metabolism”, “keg glycine serine and threonine metabolism”, and “keg cysteine and methionine metabolism”, were upregulated in Cluster 1 (Supplementary Fig. 1I). Moreover, “lipoate insertion” and “glycine metabolism” exhibited significant upregulation in Cluster 1 (Supplementary Fig. 1 J). Consequently, patients with NASH in Cluster 1 exhibited more severe disease manifestations than those in Cluster 2.

Given the substantial disparities between the two clusters, the co-expression network analysis (soft threshold = 2) was conducted to identify differentially expressed genes (Supplementary Figs. 2A and B). The yellow module, consisting of 217 genes, exhibited the strongest positive correlation with C1 (Supplementary Fig. 2C). The yellow module genes were significantly enriched in “chemokine receptors bind chemokines”, “IL-18 signaling pathway”, “regulation of response to wounding”, and “cellular response to tumor necrosis factor” (Supplementary Figs. 2D and E). Additionally, among the diseases affected by genes in the yellow module, “inflammation”, “chronic liver disease”, and “fibrosis” rank high (Supplementary Fig. 2F).

### Upregulation of three MRGs in NASH

**Fig. 10:**
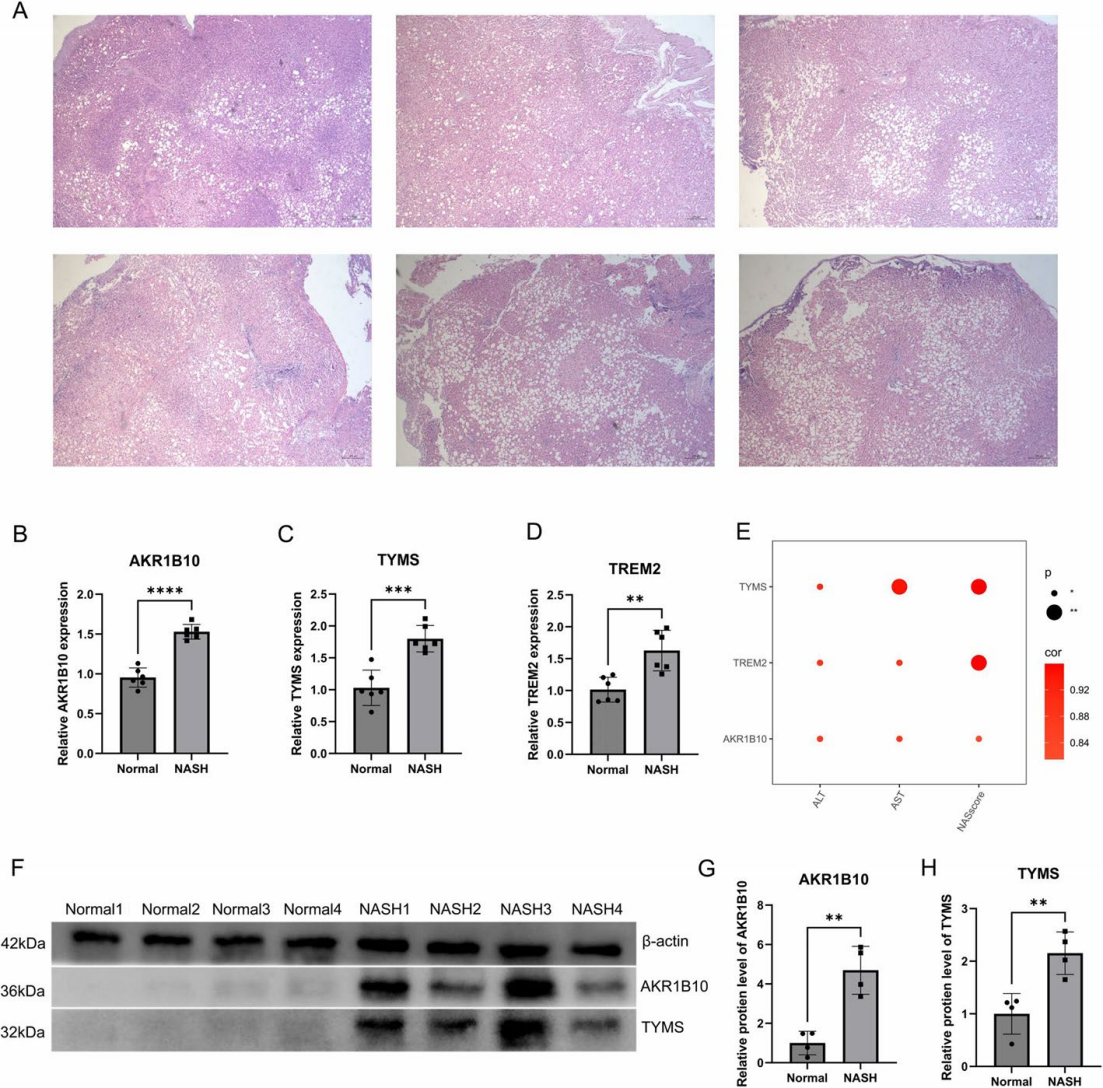
Upregulation of the three MRGs in NASH. **A** Hematoxylin and eosin staining of liver images from six morbidly obese patients, all exhibiting NAS exceeding 4. **B**-**D** At the mRNA expression level, these three genes are significantly upregulated in NASH. **E** Three MRGs exhibit significant positive correlations with AST and ALT levels, as well as the liver NAS score. **F** At the protein expression level, these three genes are significantly upregulated in NASH. **G**-**H** Quantitative visualization of WB results by generating bar graphs

Six liver specimens obtained from morbidly obese patients were subjected to hematoxylin and eosin staining, revealing a NAS exceeding 4, indicating NASH (Fig. 10A). At the mRNA level, the three MRGs were significantly upregulated in these patients (Figs. 10B-10D). Furthermore, their mRNA expression levels were positively correlated with AST and ALT levels in the blood and NAS levels in the liver (Fig. 10E). For the western blot analysis, four liver samples from healthy individuals and four samples from patients with NASH were selected. The protein expression levels of *AKR1B10* and *TYMS* mirrored their mRNA levels, and both were upregulated in NASH cells (Fig. 10F). Additionally, quantitative visualization of Western blot results through bar graphs reveals significant upregulation of *AKR1B10* and *TYMS* proteins in NASH (Fig. 10G).

## Discussion

The rising prevalence of obesity has led to a surge in the incidence of metabolic disorders associated with obesity in patients with NAFLD [38]. NASH, a severe subtype of NAFLD marked by inflammatory cell infiltration and lipid deposition, can escalate to cirrhosis, liver fibrosis, and HCC if left unchecked [39]. Given the complexity of NASH treatment, no definitive therapy is currently available. Studies have underscored the pivotal role of mitochondrial dysfunction in NAFLD pathogenesis [40]. Hence, identifying MRGs crucial for NASH may be crucial for NASH diagnosis and treatment.

In this study, 15 MRGs were observed to be differentially expressed. These genes play significant roles in cholesterol, fatty acid, and monocarboxylic acid metabolism in NASH [41–44]. These 15 MRGs are involved in various metabolic pathways that lead to NASH development. To delve deeper into the significance of mitochondrial genes in NASH, 134 machine-learning combination algorithms were employed to filter the 15 genes from the training dataset. Among these, the RF algorithm emerged as the most effective, identifying the minimum number of genes (*AKR1B10*, *TYMS*, and *TREM2*) and yielding the most accurate predictive model for NASH diagnosis. Compared to the other 133 algorithms, the predictive model generated by the RF algorithm demonstrated the highest diagnostic accuracy for NASH across both the training and eight external testing datasets.

Unlike other NASH cohorts, GSE55645 contains data from NASH blood samples. A predictive model using patient blood information precisely predicted NASH across diverse patient populations (AUC > 0.7), underscoring the promising clinical utility of this predictive model. NASH can be predicted accurately by collecting blood samples from patients. Furthermore, compared to an invasive liver biopsy, using this predictive model to analyze a small blood sample from patients significantly enhances patient compliance and boosts the detection rate of NASH. Patients with NASH can be classified into two groups based on their disease activity levels. The C1 group exhibited more severe disease, showing higher NAS, more pronounced inflammatory infiltration, increased lipid deposition, and elevated levels of proinflammatory M1 macrophages than those in the C2 group.

*AKR1B10*, which is pivotal for the metabolism of various aldehydes and ketones, is crucial for the metabolism of endogenous and exogenous carbonyl compounds [45]. *TYMS*, also known as thymidylate synthase, encodes an enzyme pivotal to DNA synthesis [46]. *TREM2* encodes a membrane receptor protein predominantly expressed on the surfaces of human monocytes, macrophages, and dendritic cells. *TREM2* affects regulating cell migration and phagocytosis, thereby influencing inflammatory and immune responses [47]. In this study, the three MRGs were upregulated in patients with NASH compared with those in healthy individuals. Moreover, compared to liver fibrosis stages F0 to F2, *AKR1B10*, and *TYMS* exhibited increased expression in fibrosis stages F3 to F4. Additionally, *AKR1B10* and *TYMS* were upregulated in individuals with HCC compared to those with NAFLD. Thus, the upregulation of the three MRGs may contribute to the progression of liver fibrosis and HCC development.

When these three MRGs are upregulated in the hepatic tissue, they promote inflammatory infiltration and lipid synthesis, exacerbate liver fibrosis, activate pro-inflammatory M1 macrophages, and inhibit fatty acid beta-oxidation and anti-inflammatory M2 macrophages [48–51]. In NASH, *AKR1B10* upregulation may lead to mitochondrial dysfunction, disrupt redox reaction balance, and cause excessive reactive oxygen species production, thereby inducing oxidative hepatocyte damage [52]. *TYMS* upregulation may lead to reduced adenosine triphosphate synthesis within the mitochondria, thereby affecting hepatic energy metabolism and hampering fatty acid oxidation [53]. Additionally, *TREM2* upregulation may contribute to mitochondrial dysfunction, impacting macrophage activation and function, thereby exacerbating the inflammatory response in NASH [54].

### Study strengths and limitations

This study's strength resides in employing machine learning algorithms to pinpoint three pivotal mitochondrial genes (*AKR1B10, TYMS*, and *TREM2*) implicated in NASH. Based on these three genes, patients with NASH can be categorized into two groups with different disease severity levels, aiding in the precise treatment of severe NASH lesions in clinical practice. Moreover, the development of a non-invasive diagnostic model for NASH using the RF algorithm addresses the invasive nature of liver biopsy, thereby overcoming its limitations. However, the limitations of this study were attributed to budget constraints, which prevented further experimental investigations to elucidate the specific mechanisms by which these three genes function in NASH.

## Conclusion

The clinical importance of this study resides in the accurate identification of MRGs in NASH, namely *AKR1B10, TYMS*, and *TREM2*. Their upregulation in patients with NASH promotes inflammatory infiltration, lipid accumulation, liver fibrosis, and the activation of pro-inflammatory immune cells. Moreover, based on these three genes, a non-invasive diagnostic model for NASH can be constructed using the RF algorithm, but subtyping of patients with NASH can be achieved. In clinical practice, the discoveries of this study can assist in precisely identifying and subclassifying patients with NASH, circumventing the invasiveness linked with liver biopsies. Early detection of severe cases is advantageous for preventing disease progression to cirrhosis or liver cancer by avoiding delays in diagnosis.

### Supplementary Information

**Supplementary Material 1.**

**Supplementary Material 2.**

**Supplementary Material 3.**

**Supplementary Material 4.**

**Supplementary Material 5.**

**Supplementary Material 6.**

## Data Availability

The datasets analyzed during the current study are available in the GEO repository [https://www.ncbi.nlm.nih.gov/geo/], Metascape repository [https://metascape.org/gp/index.html#/main/step1], GeneMANIA repository [https://genemania.org/], MitoCarta 3.0 repository [https://www.broadinstitute.org/mitocarta/mitocarta30-inventory-mammalian-mitochondrial-proteins-and-pathways], GSEA repository [GSEA (gsea-msigdb.org)], and immune cell signature markers repository [ThermoFisher Scientific—CN].
