# Supplementary material for: Machine learning-based algorithm identifies key mitochondria-related genes in non-alcoholic steatohepatitis
Source: Lipids Health Dis. 2024 May 8;23:137. doi: 10.1186/s12944-024-02122-z (PMC11077862; doi:10.1186/s12944-024-02122-z)
Supplement: Supplementary file 5 — Supplementary Material 5. [file 12944_2024_2122_MOESM5_ESM.docx]

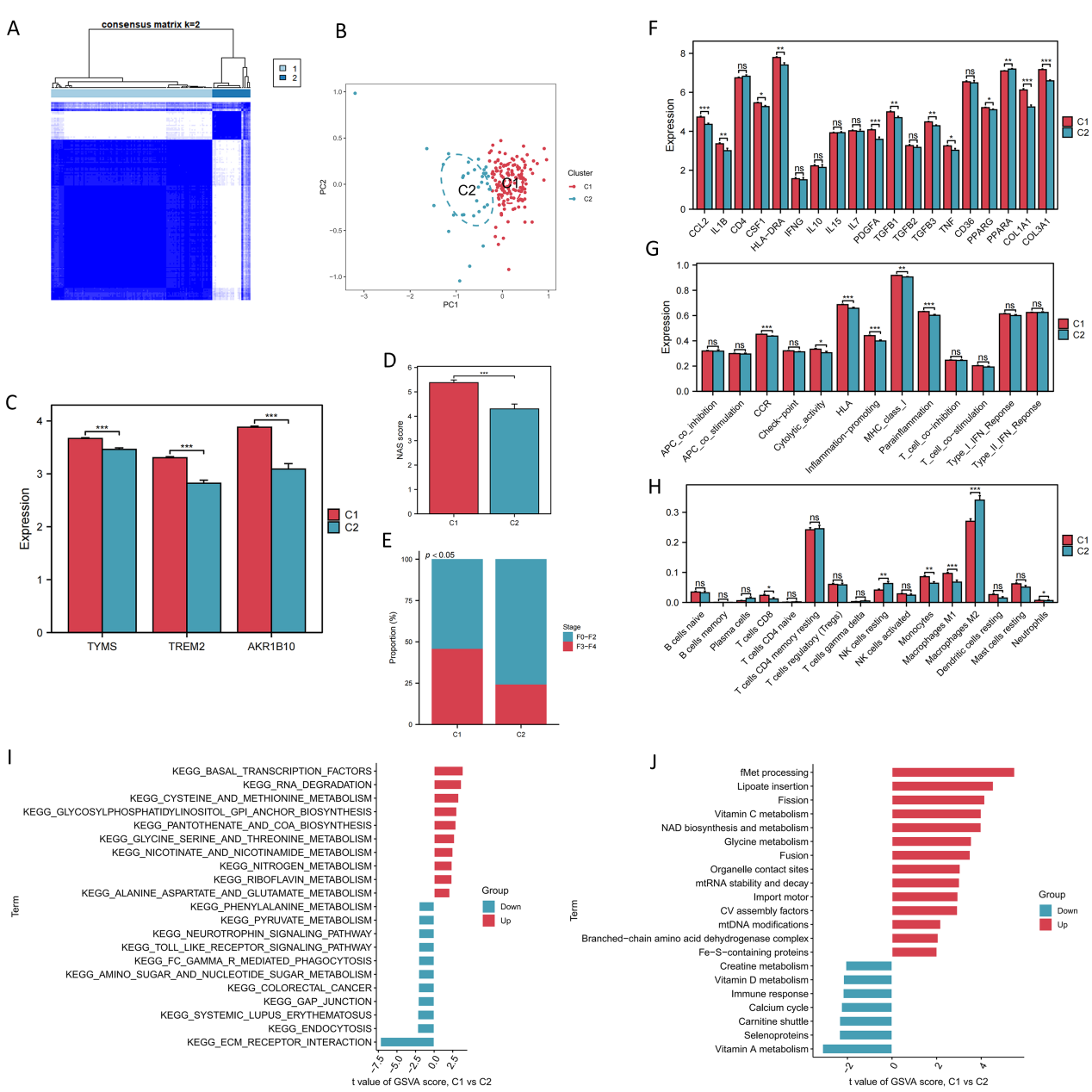


Supplementary Figure 1 The clinical and biological differences between the two groups of NASH patients. (A) NASH patients were divided into two groups. (B) NASH patients between the two groups were distinctly separated. (C) *TREM2*, *TYMS*, and *AKR1B10* were significantly upregulated in Group C1. (D) Group C1 had significantly higher NAS scores. (E) Group C1 had a higher proportion of patients in liver fibrosis stages F3-F4. (F) Pro-inflammatory genes (*CCL2, IL1B, CSF1, HLA-DRA, PDGFA, TGFB1, TGFB3, TNF*), lipid synthesis genes (*PPARG*), and liver fibrosis-related genes (*COL1A1, COL3A1*) were significantly upregulated in Group C1. (G) Immune functional scores associated with inflammation promotion were upregulated in Group C1. (H) In group C1, the proportion of pro-inflammatory M1 macrophages is higher, whereas the proportion of anti-inflammatory M2 macrophages is lower. (I) Compared to the C2 group, significant upregulated and downregulated biological pathways are observed in the C1 group. (J) Compared to the C2 group, significant upregulated and downregulated mitochondrial pathways are observed in the C1 group.


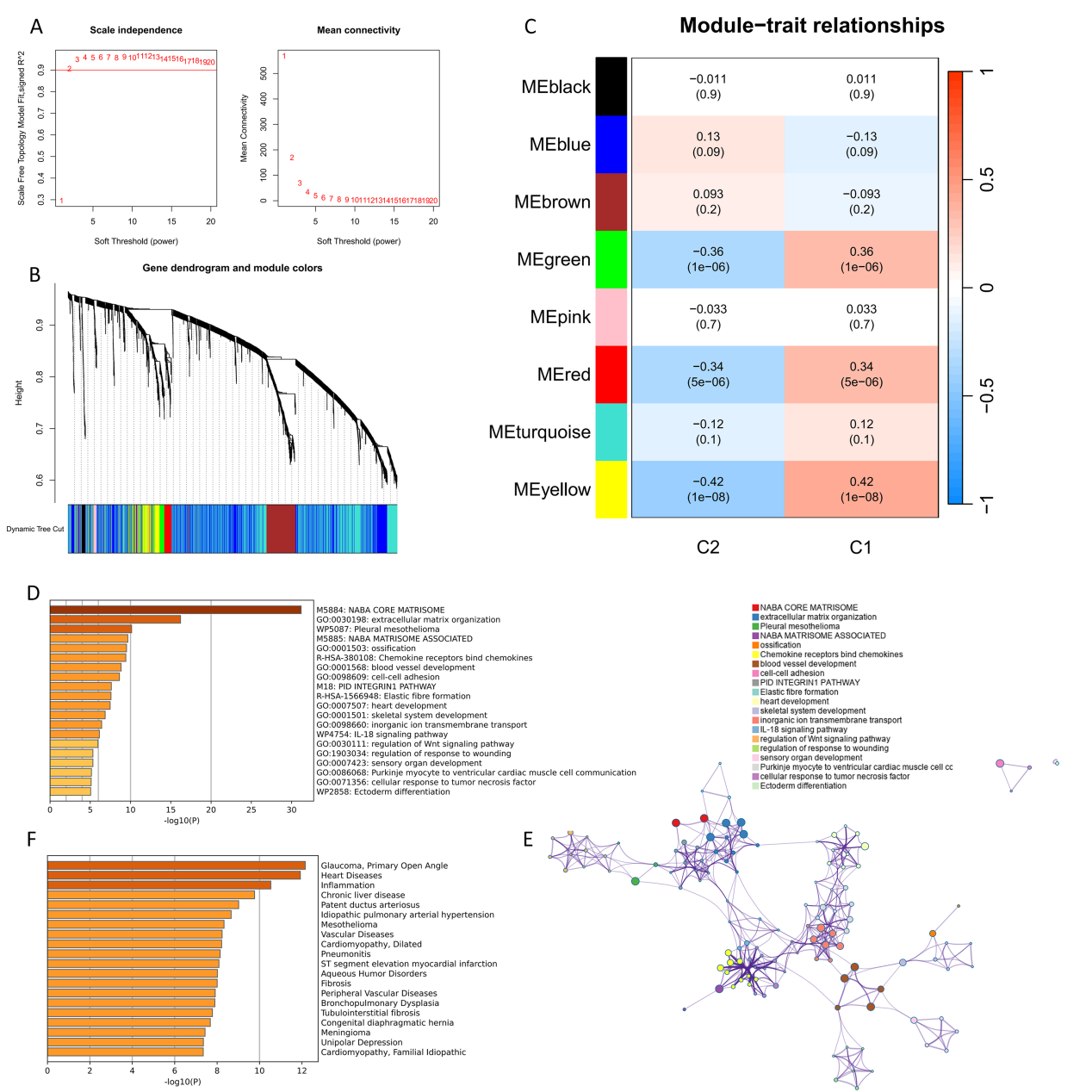


Supplementary Figure 2 271 differentially expressed genes between the two groups were identified by WGCNA analysis. (A) The soft threshold was set to 2. (B) All genes were grouped into different gene modules. (C) The 271 genes contained within the yellow module were the most significantly different genes between the two groups. (D-E) Biological pathways significantly enriched with the 271 differentially expressed genes. (F) Diseases significantly enriched with the 271 differentially expressed genes.
