# Supplementary figures and images for: Machine learning-based algorithm identifies key mitochondria-related genes in non-alcoholic steatohepatitis
Source: Lipids Health Dis. 2024 May 8;23:137. doi: 10.1186/s12944-024-02122-z (PMC11077862; doi:10.1186/s12944-024-02122-z)

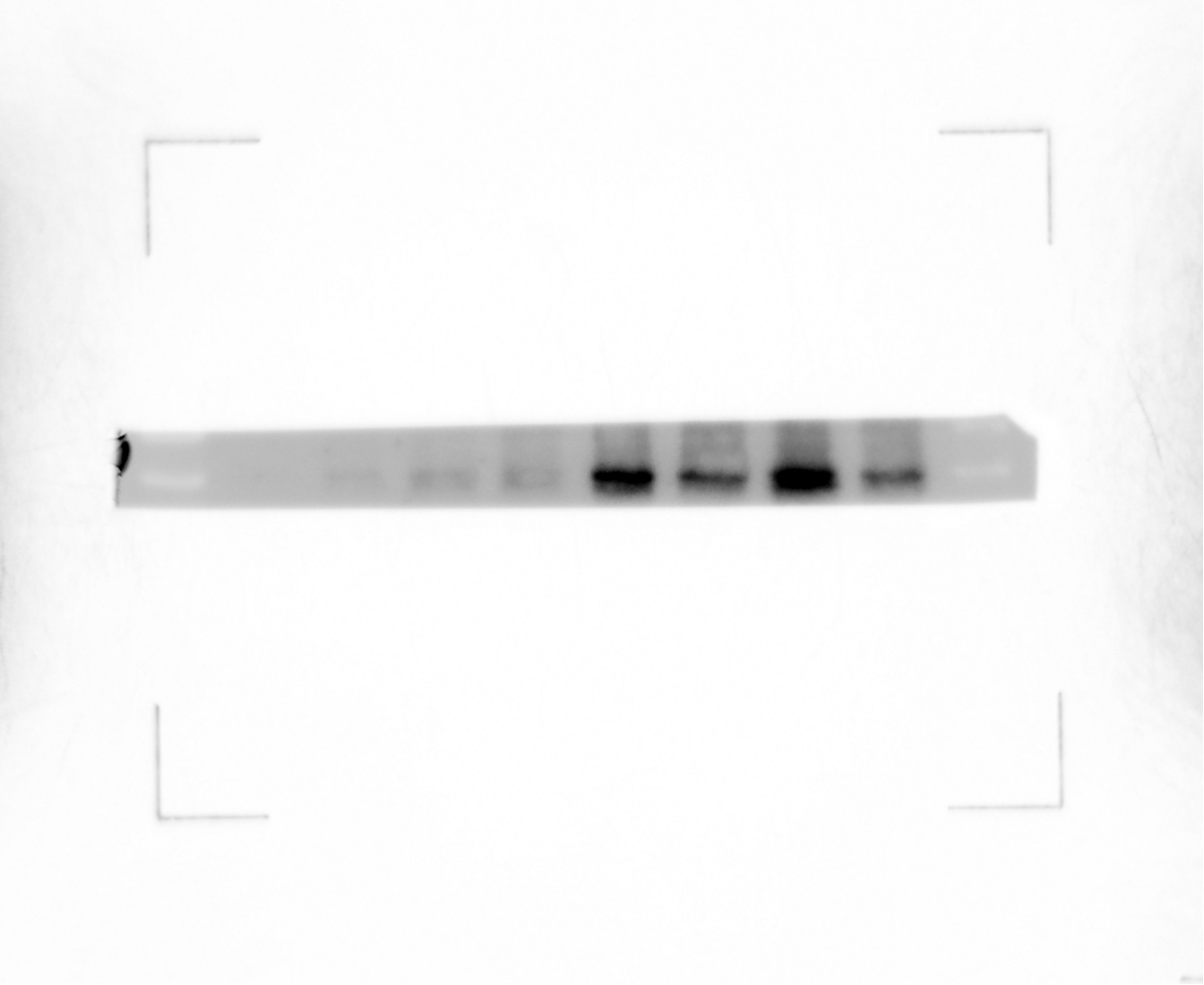

Supplement: Supplementary file 6 — Supplementary Material 6. [file 12944_2024_2122_MOESM6_ESM.zip › WBAK1B10.jpg]

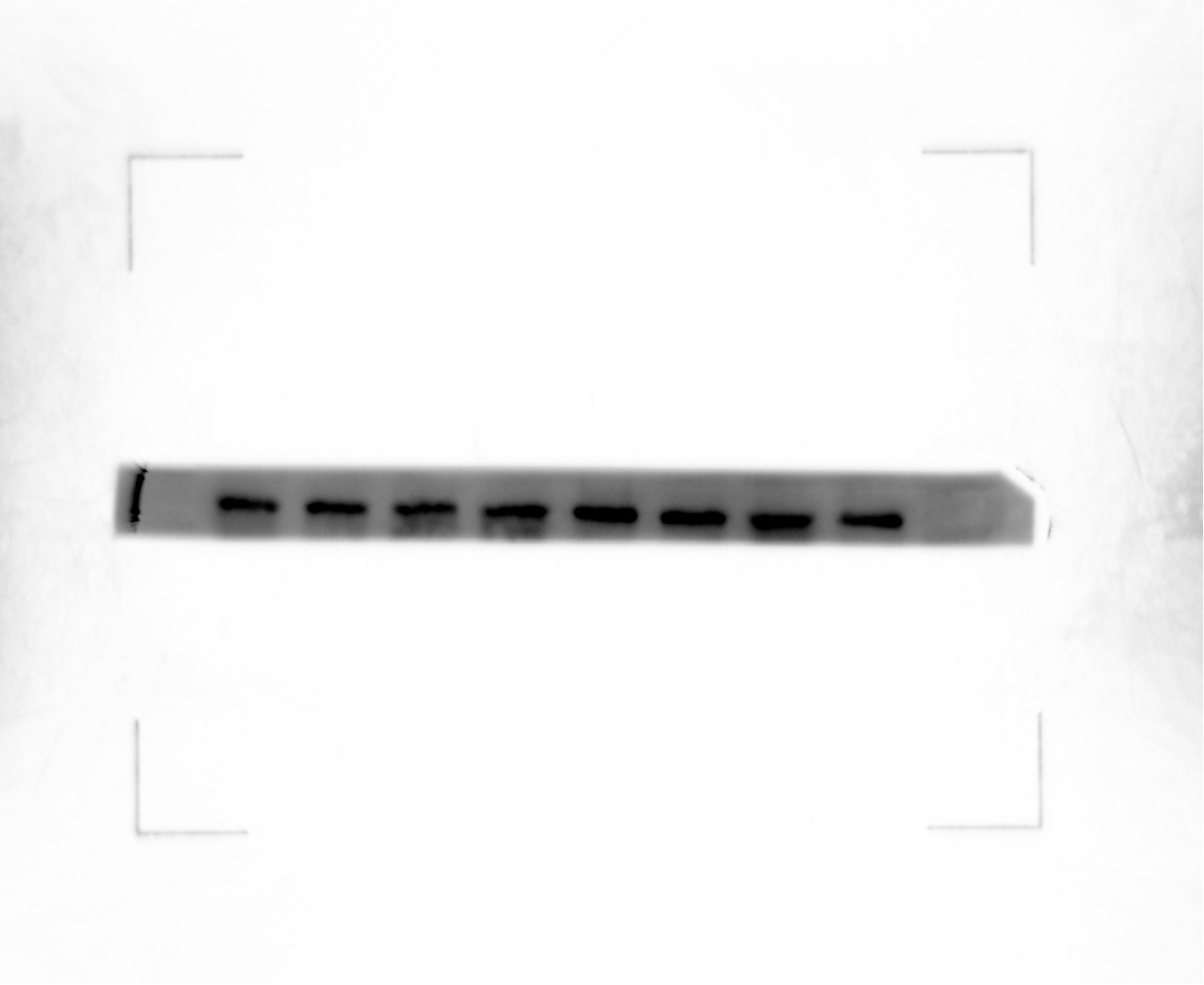

Supplement: Supplementary file 6 — Supplementary Material 6. [file 12944_2024_2122_MOESM6_ESM.zip › WBßActin.jpg]

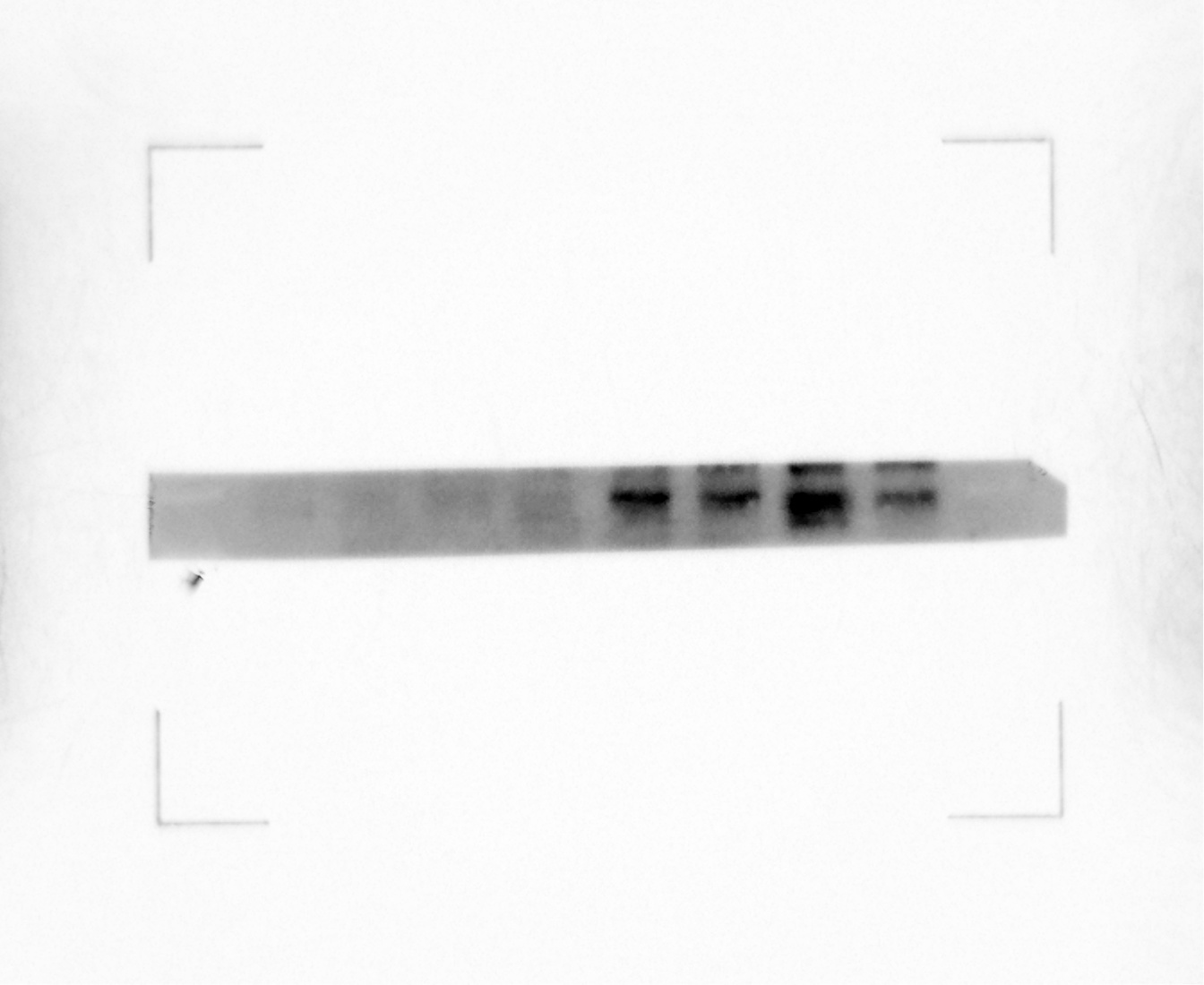

Supplement: Supplementary file 6 — Supplementary Material 6. [file 12944_2024_2122_MOESM6_ESM.zip › WBYMS.jpg]
